# Background Synaptic Activity in Rat Entorhinal Cortex Shows a Progressively Greater Dominance of Inhibition over Excitation from Deep to Superficial Layers

**DOI:** 10.1371/journal.pone.0085125

**Published:** 2014-01-15

**Authors:** Stuart David Greenhill, Sophie Elizabeth Lyn Chamberlain, Alex Lench, Peter Vernon Massey, Kathryn Heather Yuill, Gavin Lawrence Woodhall, Roland Spencer Gwynne Jones

**Affiliations:** 1 Department of Pharmacy and Pharmacology, University of Bath, Claverton Down, Bath, United Kingdom; 2 Aston Brain Centre, School of Life and Health Sciences, Aston University, Birmingham, United Kingdom; 3 School of Biomedical & Healthcare Sciences, Plymouth University Peninsula Schools of Medicine and Dentistry, Plymouth, United Kingdom; University of Alberta, Canada

## Abstract

The entorhinal cortex (EC) controls hippocampal input and output, playing major roles in memory and spatial navigation. Different layers of the EC subserve different functions and a number of studies have compared properties of neurones across layers. We have studied synaptic inhibition and excitation in EC neurones, and we have previously compared spontaneous synaptic release of glutamate and GABA using patch clamp recordings of synaptic currents in principal neurones of layers II (L2) and V (L5). Here, we add comparative studies in layer III (L3). Such studies essentially look at neuronal activity from a presynaptic viewpoint. To correlate this with the postsynaptic consequences of spontaneous transmitter release, we have determined global postsynaptic conductances mediated by the two transmitters, using a method to estimate conductances from membrane potential fluctuations. We have previously presented some of this data for L3 and now extend to L2 and L5. Inhibition dominates excitation in all layers but the ratio follows a clear rank order (highest to lowest) of L2>L3>L5. The variance of the background conductances was markedly higher for excitation and inhibition in L2 compared to L3 or L5. We also show that induction of synchronized network epileptiform activity by blockade of GABA inhibition reveals a relative reluctance of L2 to participate in such activity. This was associated with maintenance of a dominant background inhibition in L2, whereas in L3 and L5 the absolute level of inhibition fell below that of excitation, coincident with the appearance of synchronized discharges. Further experiments identified potential roles for competition for bicuculline by ambient GABA at the GABA_A_ receptor, and strychnine-sensitive glycine receptors in residual inhibition in L2. We discuss our results in terms of control of excitability in neuronal subpopulations of EC neurones and what these may suggest for their functional roles.

## Introduction

The entorhinal cortex (EC) acts as a dynamic processor of information entering and leaving the hippocampus. The lamina and columnar structure of the EC provides a means of internal integration of information processing, and organization of its inputs and outputs determines its associative interactions with the rest of the neuraxis. The perforant path provides the major source of hippocampal input, projecting from layer II (L2; principally to the dentate gyrus and CA3) and layer III neurones (L3; principally to CA1 and the subiculum). These neurones receive convergent input from higher order cortices directly, and via adjacent cortices (presubiculum, perirhinal, parahippocampal). Hippocampal output is directed back to the neocortex via projections from CA1 and subiculum to neurones in layer V (L5) of the EC. In addition, the deeper neurones have associative connections with neurones in the superficial layers, and these provide a substrate for reverberant activity, which may be involved in reinforcement of stored information. The intimate association of the EC and the hippocampus probably indicates a complementary role of these areas in memory processes [1]–[2].

Increasing attention is being paid to the crucial role of the EC in spatial memory, in particular, in spatial representation and navigation [3]. To navigate, most animals derive spatial cues from external landmarks, combining this with computation of position from motional self-location cues. Principal neurones in the hippocampus may function as place cells to fulfill the former function, whilst grid cells, head-direction cells, and conjunctive cells (both grid and head-direction properties) in other areas, including the EC, may contribute to the latter [3]. Within the EC there appears to be a lamina-based gradation of function, with L2 cells biased towards grid function, those in L3 towards head-direction, and L5 towards conjunctive function.

Other studies point to a lamina-specific delineation of functional responsiveness within the EC. For example, slow-wave oscillations (akin to cortical up-down states) in the EC are prominent in L3, but similar activity is weaker in L2, and L5 is reluctant or unable to participate in such activity [4]. Pharmacologically-induced gamma-frequency oscillation are also more pronounced in L3 compared to deeper and more superficial layers [5], whereas theta oscillatory activity is more prominent in L2, than in L3 or L5 [6].

EC dysfunction has often been implicated in neurological disorders particularly temporal lobe epilepsy (TLE) [7]–[15]. *In vitro* experiments in rat brain slices have demonstrated a pronounced susceptibility of the EC to acutely provoked epileptogenesis [16]–[23]. Pharmacologically induced seizures arise predominantly in the EC and propagate to adjacent cortical and hippocampal areas [16], [20], [24]–[27], but within the EC, acute epileptiform activity appears to be initiated in deep layers of the EC [16], [20], [21], [27], [28] leading to the suggesting that deep layers may be more susceptible to pathological synchronization [20], [21], [29].

The functional roles played by subpopulations of neurones in integrative processing and pathological activity will be dependent on many factors, including their intrinsic properties and connectivity within internal and external networks. Increasing attention has been paid to the role of background synaptic activity in modulating the properties of cortical neurones and determining input and output responsiveness. Individual cortical neurones are synaptically targeted by thousands of inputs derived from both excitatory (glutamate) and inhibitory (GABA) neurones in a dense and complex network. Both transmitters are continuously released by action potentials within network interconnections and by activity-independent release (miniature events). This background synaptic activity is a reflection of the moment-to-moment state of the network and is suggested to be instrumental in determining the excitability of any given neurone. It provides a source of stochastic resonance enhancing gain and signal detection [30]–[34]. Such activity may play a major role in shaping the processing of entrant, reentrant and afferent information in the EC.

One way of studying background synaptic activity is to monitor spontaneous excitatory and inhibitory currents using whole cell patch clamp recording, and we have adopted this approach in the EC to make qualitative comparisons of background inhibition and excitation in L5 and L2 neurones [35]–[38]. In the present investigation we have extended these observations to include L3 pyramidal neurones. However, there are a number of technical limitations with this approach (see methods), and, more recently, we have employed an analytical method to estimate global background synaptic conductances from measurement of fluctuations in membrane potential (termed VmD) derived from sharp electrode, intracellular recordings [39]–[44]. In the current investigation we have used both whole cell patch clamp recording, and VmD estimations to compare fast background inhibition mediated via GABA_A_-receptors (GABA_A_r) and excitation mediated by AMPA-receptors (AMPAr) in L2, L3 and L5 neurones in the EC. We have investigated how these background synaptic conductances are altered during progressive synchronization of network activity.

## Materials and Methods

### Ethics statement

All experiments were performed in accordance with the U.K. Animals (Scientific Procedures) Act 1986, European Communities Council Directive 1986 (86/609/EEC) and the University of Bath ethical review document, which requires that the number of animals used is kept to a minimum and every precaution was taken to reduce suffering and stress. At this institution, all research work involving use of animal tissue requires submission of a consideration of ethical implications form by the principal investigator. This is reviewed by a second investigator, external to the research group, and by the Head of Department, and requires signatory approval from both before being submitted to and reviewed by the Departmental Research Ethics Officer (DREO). The DREO will discuss any issues raised with the investigator. The DREO submits report to the University Ethics Committee detailing the ethical implications of all research within the Department on an annual basis. This ensures that the ethical implications of the research have been considered and that there is be a process in place for managing any ethical issues. All these processes have been adhered to in the current experimental work.

### Slice preparation

EC slices were prepared [45] from male Wistar rats (60–100 g; P28–40) anaesthetised with ketamine (120 mg/kg) plus xylazine (8 mg/kg). Rats were decapitated and the brain removed and immersed in artificial cerebrospinal fluid (aCSF; see below for composition) at 4°C. Slices (400 µM) were cut using a Campden Vibroslice and stored in aCSF bubbled with carbogen (95% O_2_/5% CO_2_) at room temperature. Because of the orientation of the cutting [45], ours slices were largely restricted to more ventral locations, so dorso-ventral variation in intrinsic properties of neurones was minimized. To increase neuronal survival and viability, ketamine (4 µM) and indomethacin (45 µM) were included in the cutting solution and the antioxidants, n-acetyl-l-cysteine (6 µM) and uric acid (100 µM), added to both cutting and storage solutions. We have established (Woodhall, G.L. and Jones, R.S.G., unpublished observations) that the use of additives during cutting and storage produces robust and long-lasting slices, but does not have any apparent effect on the pharmacology of glutamate or GABA transmission. Nevertheless, in both recording situations slices were allowed to equilibrate in the recording chambers for at least 1 hour prior to recording to allow for washout of these agents. For patch clamp recordings slices were transferred to a recording chamber perfused (2 ml/min) with oxygenated aCSF at 31–32°C on an Olympus BX50WI microscope. Neurones were visualized using DIC optics and an infrared video camera. In VmD experiments slices were transferred to a recording chamber where they were held at the interface between a continuous perfusion of oxygenated aCSF (1.5 ml/min) maintained at 32±0.5°C and warm, moist carbogen gas. Intracellular recordings were made “blind” from slices visualised with a binocular microscope (Wild M8). The perfusion and storage aCSF contained (in mM): NaCl (126), KCl (3.25), NaH_2_PO_4_ (1.4), NaHCO_3_ (19), MgSO_4_ (2), CaCl_2_ (2), and D-glucose (10). For cutting the slices at 3–4°C, NaHCO_3_ was increased to 25 mM to maintain pH at acceptable levels (7.3).

### Experimental approaches

As noted, we have used two experimental approaches to examine background synaptic activity in these studies. The more traditional approach involves whole cell voltage clamp recording of spontaneous synaptic currents. This is essentially looking at background activity from a presynaptic viewpoint, comparing the probabilistic release of glutamate and GABA, and making the assumption that a higher rate of release will result in greater postsynaptic excitation or inhibition, respectively, and vice versa. There are several technical issues with this approach. Experimental conditions are tailored towards recording of either excitatory or inhibitory currents in isolation, and therefore do not take into account how alterations in network activity and the postsynaptic responses to either transmitter may be altered by changes in the other. Recordings are conducted at somatic sites and these will not adequately detect events at distal dendritic sites. This means that frequencies of IPSCs and EPSCs will not reflect a true contribution of inhibition or excitation to overall cellular activity. The differential location of excitatory and inhibitory synapses also means that synaptic excitation (distal location) may be underestimated compared to inhibition (more proximal) by this approach. Patch solutions for recording of synaptic currents generally include Na^+^ and K^+^-channel blockers to improve postsynaptic space-clamp and partially obviate some of the issues with recording resolution. However, this means that a realistic estimation of cellular excitability is impossible.

We have developed a complementary approach based on what is referred to as the VmD method. This was developed by Alain Destexhe and colleagues [42]–[44], using the presumptive synaptic activity of model neurones to attempt to recreate the biological activity of real neurones under conditions of intense synaptic activity (referred to as a “high-conductance state” [42]–[44]). We have adapted this mathematical approach as a means of quantifying the background synaptic activity based on the ongoing biological fluctuations in real neurones. The approach essentially looks at background synaptic activity from a postsynaptic perspective. The VmD method relies on the premise that momentary fluctuation in somatic membrane potential largely reflects the combined effects of on-going global inhibitory and excitatory postsynaptic conductances, which result from the presynaptic transmitter release occurring in response to network activity. Although this method was developed and tested using theoretical and experimental approaches applicable to high conductance *in vivo* network states, we have experimentally and pharmacologically validated its use to estimate background conductances in the relatively quiescent conditions extant in EC slices, and, subsequently, employed it to determine the effects of diverse anticonvulsants on these conductances [39]–[41]. There are potential errors and limitations that have to be considered in terms of establishing absolute levels of background conductances, particularly membrane potential fluctuations arising from intrinsic membrane ion channels [42]. However, since we are looking principally at relative differences within and between neurones, these are not prohibitive issues, and it provides an excellent way to complement studies of presynaptic release with overall postsynaptic activity.

### Whole-cell patch clamp recordings of transmitter release

Patch pipettes pulled from borosilicate glass were used for recording spontaneous or miniature excitatory postsynaptic currents (sEPSCs and mEPSCs, respectively). They were filled with a Cs-gluconate based solution containing (in mM) D-gluconate (100), HEPES (40), QX-314 (1), EGTA (0.6), MgCl_2_ (5), TEA-Cl (10), phosphocreatinine (5); ATP-Na (4) and GTP-Na (0.3). To record spontaneous or miniature inhibitory PSCs (sIPSCs and mIPSCs), the patch solution contained CsCl (100), HEPES (40), QX-314 (1), EGTA (0.6), TEA-Cl (10), MgCl_2_ (5), ATP-Na (4) and GTP-Na (0.3). Solutions were adjusted to 275 mOsmol and pH 7.3 with CsOH. Whole-cell voltage clamp recordings (holding potential −60 mV) were made from pyramidal neurones in L3 of the medial division of the EC, using an Axopatch 200B amplifier. Signals were filtered at 2 kHz and digitized at 20 kHz. Series resistance compensation was not employed, but access resistance (10–30 MΩ) was monitored at regular intervals and cells were discarded if it changed by more than ±10%. Liquid junction potentials (EPSC +12.0 mV; IPSC +10.2 mV) were estimated using pClamp-8 software, and compensated for in the holding potentials. When recording IPSCs, AMPA-receptors and NMDA-receptors were blocked with bath applied NBQX and 2-AP5, respectively.

Data were recorded using Axoscope software, and Minianalysis (Synaptosoft, Decatur) was used for analysis of PSCs off-line. Spontaneous events were detected using a threshold-crossing algorithm. Cumulative probability distributions of interevent interval (IEI) were compared using the Kolmogorov-Smirnoff test (KS). When data were pooled for this analysis, a minimum of 200 events was sampled during a continuous recording period for each neurone under each condition. Mean amplitudes, rise times (10–90%) and total decay times were compared using a t-test.

### VmD estimations of postsynaptic background conductances

Sharp electrodes pulled from borosilicate glass and filled with potassium acetate (3M, pH adjusted to 7.3); tip resistances of 80–120 MΩ) were used to make intracellular voltage recordings using an Axoprobe 1A amplifier (Molecular Devices). When membrane potential had stabilised after impalement, estimates of global background excitation (E_bg_) and inhibition (I_bg_) were derived from membrane potential fluctuations at regular intervals throughout the recordings using the VmD method. This approach was derived by Rudolph *et al.*, [42] and we have adapted it for recording in EC slices [39]–[41]. Precise technical details of the approach are available in these papers [39], [42], and are not repeated here. Briefly, neurones were depolarised (for 15–20 s) by injection of two levels of known positive current via the recording electrode. The values of the currents differed from neurone to neurone, but were maintained the same throughout any individual experiment. One level was chosen to elicit a depolarization to within 1–2 mV of action potential threshold, and the second was adjusted to depolarize the neurone to about half way between this and resting membrane potential. Membrane potential fluctuations at these two levels were fitted to Gaussian distributions (Prism 4 software, GraphPad, San Diego, USA) and the mean and variance of the membrane potential determined. Leak conductance in each neurone was calculated from the ohmic response produced by a small (0.1 nA 100 ms) hyperpolarizing current, injected at resting membrane potential. These parameters, together with mean reversal potentials for AMPA-receptors and GABA_A_r mediated synaptic responses ([39] plus unpublished data), allowed us to use the VmD relationship to quantitatively estimate background inhibitory and excitatory conductances resulting from global network input onto individual neurones. Statistical analysis (paired *t*-tests or one-way ANOVA) was performed with Prism 4 software. All error values in the text refer to standard error of the mean.

Cellular excitability was determined by injecting depolarizing current pulses at resting potential. Action potential (AP) thresholds with respect to resting membrane potential were determined using brief incremental peri-threshold injections of depolarising current (0.1–1.0 nA, 50 ms) via the recording electrode. Trains of action potentials were also elicited by longer, supra-threshold current pulses (0.2–1.0 nA, 200 ms), and the number of spikes per pulse determined.

In some experiments, designed to compare the effects of blocking GABA inhibition in L2, we employed extracellular recording of spontaneous epileptiform activity. In these studies we used patch pipettes (filled with normal ACSF, and broken back to give tip resistances of around 1–5 MΩ) and an NPI EX-10C differential amplifier for recording local field potential activity. In some cases simultaneous recordings were made in different layers.

### Materials

Salts used in preparation of aCSF and electrode solutions were purchased from Merck/BDH or Fisher Scientific (UK). Indomethacin, n-acetyl-l-cysteine and uric acid were purchased from Sigma (UK). Ketamine was supplied by Fort Dodge Animal Health Ltd (Southampton, UK) and xylazine by Bayer AG (Leverkusen, Germany). Drugs were applied by bath perfusion. The following drugs were supplied by Tocris UK or Ascent Scientific UK: 2-AP5 (D-2-amino-5-phosphonopentanoic acid), NBQX (6-nitro-7-sulphamoylbenzo[f]quinoxalone-2,3-dione disodium), bicuculline methiodide, QX-314 (N-(2,6-Dimethylphenylcarbamoylmethyl) triethylammonium chloride). Picrotoxin and strychnine HCl were purchased from Sigma UK.

## Results

### Lamina comparison of spontaneous GABA and glutamate release

Previous work from this laboratory has detailed characteristics of s/mEPSCs and s/mIPSCs in L2 and L5 of the EC [35]–[38]. To complete the laminar comparison of spontaneous release of glutamate and GABA across the EC, we have made similar studies in L3 and we present representative data (from 19 neurones each for sEPSCs and sIPSCs) here. We stress that some of the data are from previously published studies [35], [38] but a summary (supplemented by some additional recordings) is presented here for direct comparative purposes.

#### EPSCs

**Figure 1:**
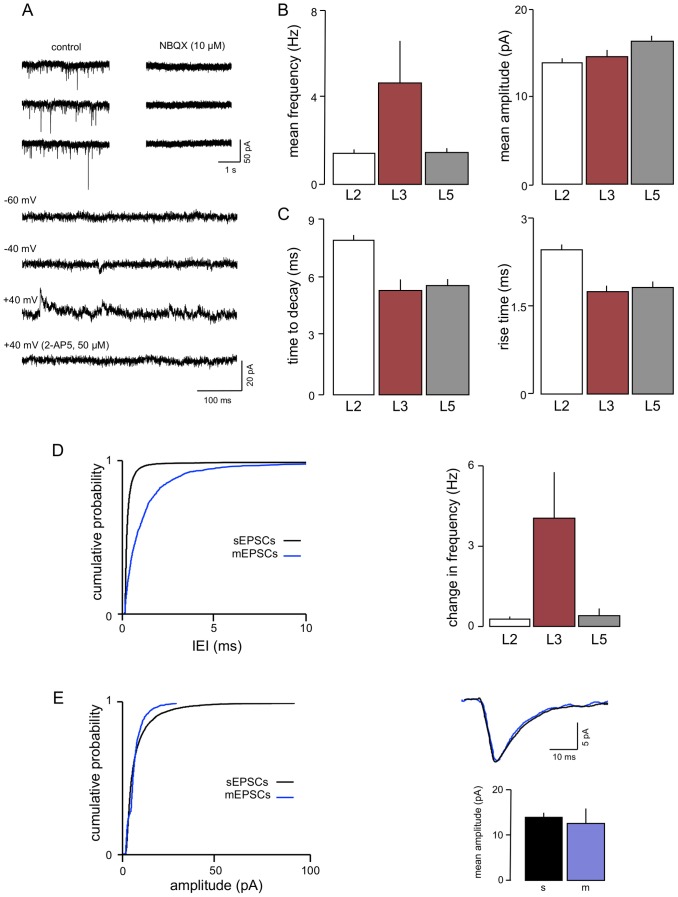
Characteristics of EPSCs in L3. A. Voltage clamp recordings from one neurone show that the AMPAr antagonist NBQX essentially abolished sEPSCs. At more positive holding potentials, occasional, slow sEPSCs were detected that were mediated by NMDAr, as they were blocked by 2-AP5. B. A comparison of sEPSC properties Showed that the mean frequency of events in L3 was greater than that in either L2 or L5. Amplitudes were similar across layers, but kinetics (C) were slower in L2 compared to the deeper layers. D. Action potential driven release accounts for a much higher proportion of spontaneous excitation in L3. Cumulative probability analysis of data pooled from 10 neurones (graph on left) showed a large increase in IEI (shift to the right) in L3 that was substantially greater than that seen in L2 or L5. This is very clearly illustrated by the bar graphs (right) showing the accompanying change in frequencies (sEPSC frequency minus mEPSC frequency). E. Neither amplitude nor kinetics of EPSCs in the same neurones were significantly different in the presence of TTX (mEPSCs shown in blue).

The properties of EPSCs in the 3 layers are summarized in Figure 1. As in L2 and L5 [35], the vast majority of sEPSCs were mediated via AMPAr (Figure 1A). Thus, spontaneous currents were essentially undetectable in the presence of AMPAr antagonists (NBQX, SYM 2206, or GYKI 53655) at a holding potential of −60 mV. However, at more positive holding potentials, occasional small slow events could be detected; these reversed at around 0 mV and were blocked by 2-AP5, indicating that in this layer, as in L2 and L5 [35], sEPSCs mediated by NMDA receptors were evident at a very low frequency.

The mean IEI of sEPSCs in L3 was 198±28 ms, corresponding to a mean frequency of 5.1±1.1 Hz, which is considerably higher than that of sEPSCs in either L5 (714±39 ms) or L2 (756±28 ms) (Figure 1B; cf. [38]). In contrast, the mean amplitude (Figure 1C) in L3 (14.1±0.7 pA) did not differ greatly from the other layers, although it was slightly greater than that in L2 (13.2±0.3 pA) and less than that in L5 (15.7±0.5 pA). Rise and decay kinetics (Figure 1D) in L3 (1.8±0.2 ms and 5.9±0.5 ms, respectively) were similar to those recorded for sEPSCs in L5 (1.9±0.1 ms and 5.7±0.3 ms; [35]), but both parameters were slower in L2 (2.5±0.1 ms and 8.0±0.4 ms; [35]). Previously, we have also shown that neurones in all layers are not particularly electronically compact, with estimated electrotonic lengths of 2.5 and 1.6 in L5 and L2, respectively [35]. The value estimated for L3 (1.6; [46]) was the same as that in L5, so it is possible that the faster kinetics of sEPSCs in L3 and L5 may be associated with the shorter electronic lengths of the neurones in these layers compared to L2.

There was a clear difference between L3 and L2 or L5 in the contribution of activity-dependent release to overall spontaneous release (Figure 1E). Thus, in L3 neurones, application of TTX (1 µM; n = 8) caused a 6-fold increase in IEI from 220±28 ms to 1284±243 ms, reflecting a decrease in frequency from 6.4±1 Hz to 1.0±0.3 Hz. The mean amplitude of events (Figure 1E) was slightly less in the presence of TTX, but not significantly so. However when we examined the cumulative probability distributions in pooled data, it was apparent that there was a loss of some of the larger amplitude events, which produced a significant (*P*<0.01; KS) shift to the left, evident at the top end of the distribution. In contrast, in both L2 and L5, TTX only caused a 15–20% reduction in sEPSC frequency [35]. Thus, L3 neurones show a much great preponderance of action potential driven release compared to the other layers where activity-independent, monoquantal release predominates.

#### IPSCs

**Figure 2:**
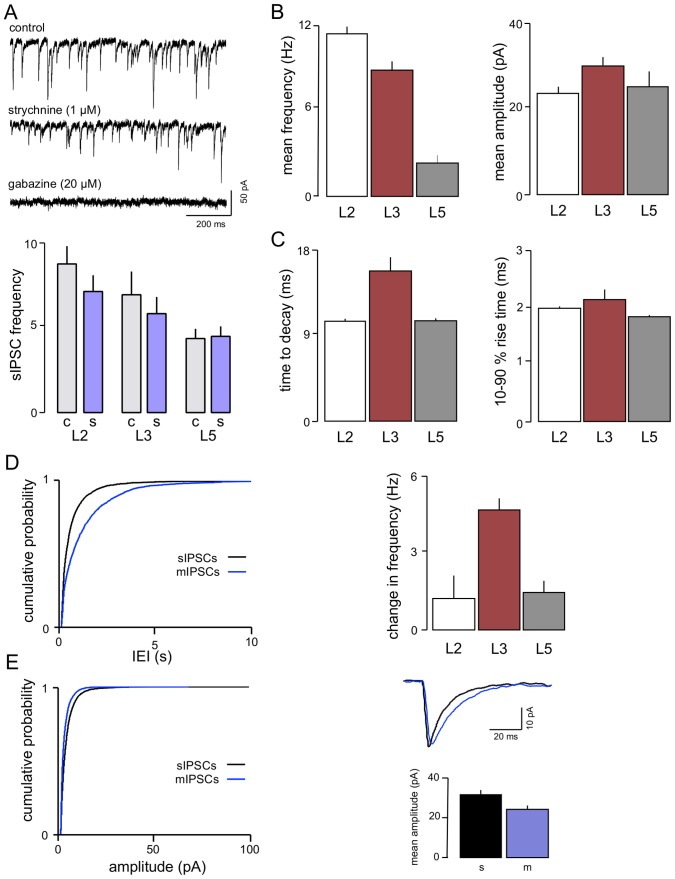
Characteristics of IPSCs in L3. A voltage clamp recordings from one neurone show that sIPSCs are largely mediated by GABA_A_r. However, there was a reduction in frequency with the glycine receptor antagonist strychnine. Although this generally only amounted to around 10–15% it was significant when assessed by a paired t-test. A similar effect was seen previously in L2 [37]. We have added a number of new recordings to this data and the reduction again just reaches significance with a paired t-test. In contrast, no effect of strychnine was seen in L5. B. sIPSC frequency was lower than in L2 but still considerably higher than in L5, whereas the both mean amplitude and decay time in L3 (C) were greater than either of the other layers (D). Action potential driven GABA release, like glutamate release (see Figure 1) was more prominent in L3 compared to L2 or L5. Thus application of TTX resulted in a marked change in frequency of events (sEPSC frequency minus mIPSC frequency). (E) In addition, the mean amplitude of events was also lower in TTX, although time to decay was slower.

IPSCs in L3 neurones were recorded at a holding potential of −60 mV, with symmetrical [Cl^−^], where they were evident as fast inward currents. In agreement with our studies in L2 and L5 [37] these were largely eliminated (n = 7) by the GABA_A_-receptor blocker, gabazine (20 µM; Figure 2A). However, the glycine receptor antagonist, strychnine, applied before gabazine (1 µM), also had a weak effect on sIPSCs reducing the frequency by around 10–20% (n = 6) without affecting amplitude (mean IEI 168±117 ms v 198±134 ms Figure 2A). KS analysis of the cumulative probability distribution of IEIs (not shown) from pooled data indicated an effect that just reached significance (P<0.01). We found a similar small reduction with strychnine in L2 neurones previously [37]. We have now added further studies to those in this previous sample and the summary data shown in Figure 2A confirm a small, but significant, decrease in frequency of sIPSCs in L2 with no effect in L5.

Overall, sIPSCs in L3 (n = 19) occurred at a high frequency (10–15 Hz; Figure 2B) reflected by a mean IEI of 112±21 ms, which is similar to that seen in L2 (IEI 87±2 ms; Woodhall et al, 2005) and much shorter than that in L5 (IEI 404±10 ms; Woodhall et al, 2005). Mean amplitude in L3 (30.9±2.0 pA; Figure 2C) was larger than that in either L2 or L5 (24.0±1.8 and 25.7±3.9 pA respectively; [37]). Mean decay time of sIPSCs in L3 was 15.8±1.5 ms, which is longer than those seen in the other layers (L2 10.5 ms; 10.6 in L5), but rise time (2.2±0.2 ms) was similar to those reported for L2 and L5 (2.0±0.03 ms and 1.9±0.03 ms, respectively; Figure 2D; [37])

The relative contribution of action potential driven and action potential-independent release was determined (Figure 2E). IEI of sIPSCs in the presence of TTX (1 µM) approximately doubled from 112±21 ms to 238±44 ms (n = 10) in L3. This represents a decrease in frequency from 15.5±2.8 Hz to 6.0±1.1 Hz, indicating that approximately half of the IPSCs are action potential-dependant. This effect is similar to that seen in L5 where the IEI more than doubled (from 368.2±13.1 ms to 979.9±31.5 ms; [37]), but contrasts markedly to L2 where TTX had little or no effect [37]. In terms of absolute numbers of sIPSCs, L3 neurones actually showed a much greater decrease than L5 in the presence of TTX, as a result of their higher baseline frequency of sIPSCs.

### EPSC to IPSC comparison

Looking at baseline levels of spontaneous synaptic activity across the three layers, there are some general and obvious differences. The most prominent difference in sEPSCs is the higher frequency noted in L3. Amplitudes are similar, with slightly larger events in L5, and in terms of kinetics the slower decay time in L2 is prominent. In the case of inhibition, the high frequency of sIPSCs in L2 and L3 compared to L5 is the most notable difference. To gain a better comparative picture of overall spontaneous synaptic activity across the three layers, we derived arbitrary charge transfer (CT) values for spontaneous synaptic activity. These values are the product of mean frequency, amplitude and decay times.

**Figure 3:**
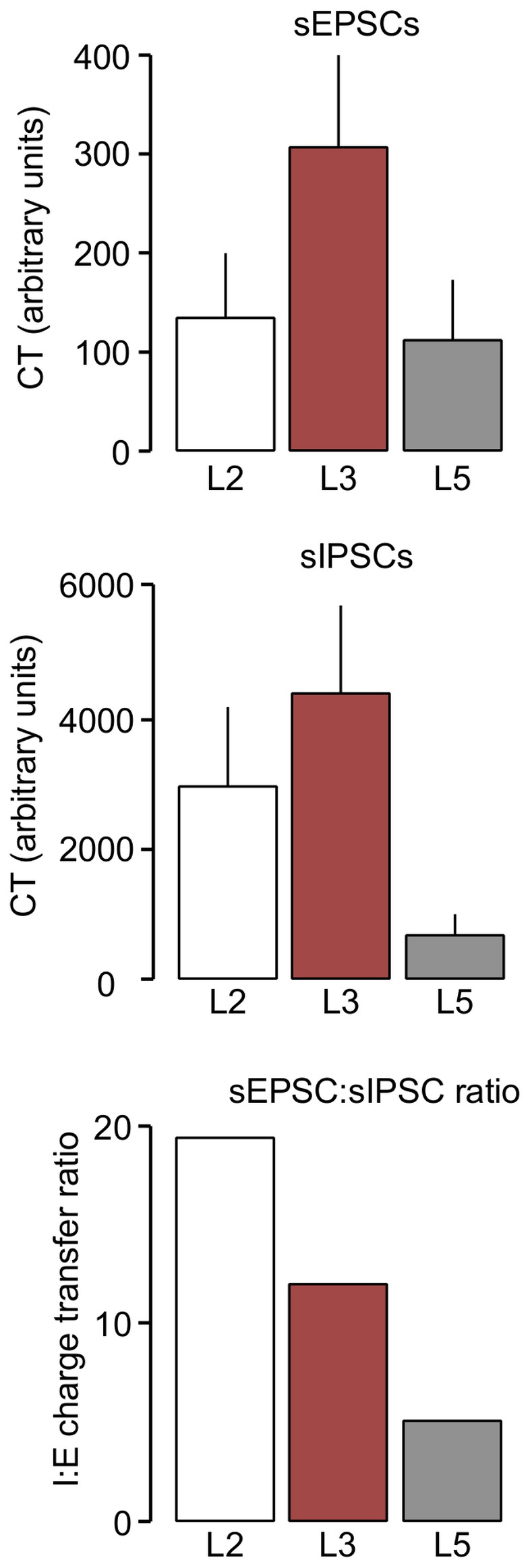
Comparison of inhibition to excitation in whole cell patch clamp studies. The total charge transfer (CT) associated with sEPSCs, was greatest in L3 and approximately equal in L2 and L5. Charge transfer of sIPSCs was also greatest in the middle layer, but substantially markedly less in L5 than the more superficial layers. The resultant ratio calculations suggested a substantial dominance of inhibition in all layers, with L2 showing by far the greatest bias and L5 the least.

For sEPSCs, CT was highest in L3 (340.3) and approximately the same in L2 (147.8) and L5 (125.3) (Figure 3 top). In the case of sIPSCs (Figure 3 middle), again L3 gave the highest CT value at 4345.2. This was followed by L2 at 2898.0 with L5 markedly lower at 653.8. Cortical neurones are under a simultaneous and continuous bombardment from both glutamate and GABA synapses. Although we have not recorded excitatory and inhibitory currents simultaneously in individual neurones, we determined the ratio of CT values as an approximate measure of the relative influence of spontaneous excitation to inhibition across the different populations of neurones. This gave inhibition∶excitation (I∶E) ratios of 19.6. in L2 followed by 12.8 in L3 and 5.2 in L5 (Figure 3 bottom). These values represent, at best, a rough comparison, as sEPSCs and sIPSCs were recorded isolated from each other, and the former were recorded with inhibition intact but the latter with excitation blocked. Nevertheless, they do indicate that overall, background inhibition predominates in all three populations, but that its relative strength is more marked in a progression from deep to superficial in the EC.

### Laminar comparison of global background synaptic activity

Recordings of sEPSCs and sIPSCs give an indication give us some idea of the relative level of inhibition and excitation exerted via the network, from a presynaptic view point and on a moment-to-moment basis. However, a more integrated assessment of the on-going inhibition and excitation seen by the postsynaptic cell and its effects on excitability requires a different approach. We have adopted the VmD method [39], [42] to assess global synaptic conductances, estimating background GABAergic inhibition and glutamatergic excitation concurrently in the same neurone. We have used this approach to complement and extend the results of our whole cell patch clamp studies, and to examine whether background network activity may be instrumental in determining the susceptibility of different populations of EC neurones to participation in synchronous activity.

**Figure 4:**
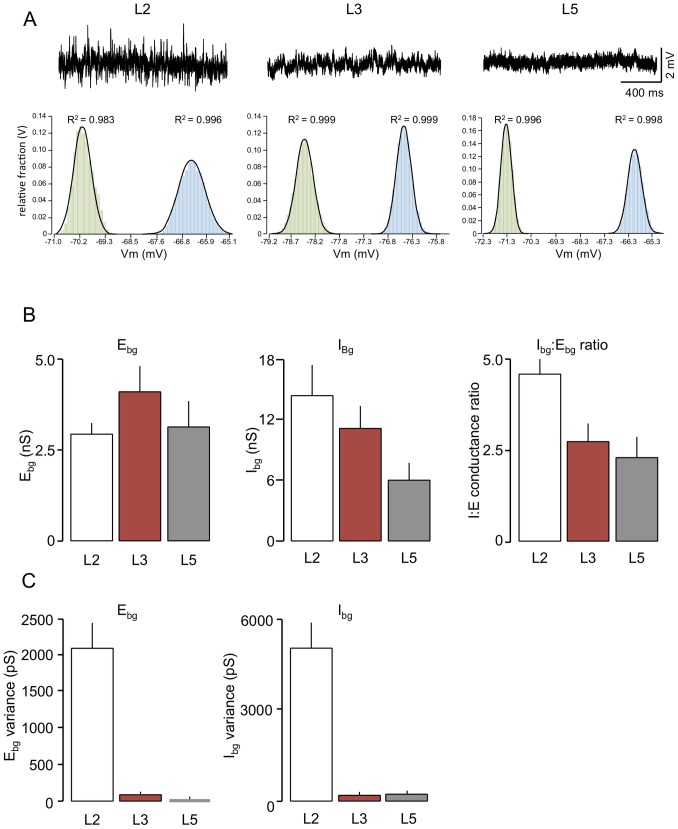
Simultaneous VmD estimations of excitatory and inhibitory background conductances. A. The raw traces show sample intracellular recordings from three neurones recorded in the same slice, and showing a great peak-to-peak voltage activity in L2. Estimation of background inhibition and excitation (see methods) required calculation of mean and variance of membrane potential at two levels of injected current (not shown). The histograms show the frequency distributions of membrane fluctuations at the two levels of injected current (geen being closer to resting potential and blue slightly more depolarized) in sample neurones from each layer. The solid lines on the graphs show the potential fluctuations constrained to a Gaussian function and in each case the goodness of fit (R^2^) was close to unity. B. Comparison of average data for background synaptic conductances estimated in the three layers of the EC. Background excitation (E_Bg_) was highest in L3 and approximately 40% higher than that in L2 or L5. Inhibition (I_Bg_) was greatest in L2 followed by L3 and L5. The resultant ratios showed a predominance of inhibition in a ll three layers, but in L2 this was this was almost double that seen in the other layers. C. The variance associated with the conductances (which is suggested to reflect the level of temporal synchrony in presynaptic inputs) was dramatically higher for both inhibition and excitation in L2 than in either of the deeper layers.

The comparative lamina data obtained using the VmD approach are illustrated in Figure 4. I_bg_, estimated in L5 neurones (n = 10; Figure 4A), was 6.1±1.7 nS. Concurrently, E_bg_ was about half this at 3.1±0.7 nS, with a mean I∶E ratio in these neurones of 2.3±0.6. Similar estimations in L3 neurones (n = 17) gave a much higher I_bg_ of 11.2±2.1 nS and a slightly greater E_bg_ of 4.1±0.7 nS, but the I∶E ratio based on these estimates was similar to that in L5 at 2.7±0.5. I_bg_, estimated in L2 neurones (14.3±2.7; n = 11), exceeded that in L3 and was substantially greater than that in L5. In contrast, E_bg_ was similar (2.9±0.3 nS) to L5 but again lower than L3. Consequently the I∶E ratio in L2 (4.6±0.4) was considerably higher than that recorded in the other layers. Comparing the data from whole cell patch clamp recordings (Figure 3), and VmD measurements (Figure 4) shows a good correlation between the levels of inhibition and excitation derived from the intracellular and whole-cell patch clamp recordings, with the standout difference between the layers being the greater preponderance of inhibition in L2.

As noted above, strychnine caused a small but significant reduction in sIPSC frequency in L2 in patch clamp recordings, so we determined its effect on I_bg_ and E_bg_ (n = 4). There was no significant change in E_bg_ with the glycine receptor antagonist (3.1±1.1 v 2.9±1.3), and although I_bg_ fell slightly (11.1±3.3 v 9.9±2.7) the change was not significant. The I∶E ratio was unaltered (3.7±0.7 v 3.3±0.7).

The VmD approach also allows us to estimate of the variance of the global background conductances. Destexhe and his colleagues [42]–[44] have suggested that conductances reflect the overall rate of release from the afferent neurones, whereas the variance may be related to the temporal correlation of the release in the presynaptic inputs. Thus, a large variance could reflect large numbers of synaptic terminals releasing transmitter synchronously or semi-synchronously [42]–[44]. However, this association is based on large numbers of synapses releasing at a high rate in an in vivo-like situation [42]–[44] and there are obvious limitations in making similar inferences from variance measures in our slices, where we are studying reduced networks (and hence synapses numbers) with a consequent reduction in release rates. Nevertheless, we did find some marked differences in mean variances in the different layers of the EC (Figure 4B). Under resting conditions, the mean variance of I_bg_ in L2 neurones was 5215±407 pS. This contrasts markedly with L3 and L5 where the mean variances were 99±49 pS and 105±27 pS, respectively. Likewise, the variance of E_bg_ was considerably greater in L2 neurones (2107±333 pS) compared to L3 (88±21 pS). The variance in E_bg_ was lowest in L5 (27±24 pS).

One possible confounding factor in comparing L2 to L3 and L5, is the heterogeneity of neurones in the former. Previous studies, including from this laboratory, have suggested that neurones in L2 with a pronounced time-dependent inward rectification (TDIR) during hyperpolarization, due to a strong expression of I_h_, are likely to be stellate in nature, whereas those that do not exhibit TDIR are more likely to present pyramidal-like morphology [19], [47]. Based on subsequent recordings in several hundred L2 neurones (R.S.G. Jones, unpublished), we have found that there is much more of a continuum within the correlation of expression of TDIR and morphological characteristics, and a demarcation of stellate v pyramidal on this basis is less clear than originally supposed. Nevertheless, we thought it worthwhile to compare background conductances in neurones with prominent TDIR, to those in which it is less evident. We used the population of neurones from the analysis above, together with a further 10 in which we just recorded baseline conductances. In these 21 neurones we classified 15 with clear TDIR and 6 where TDIR was minimal (non-TDIR). Mean I_Bg_ was 15.1±3.6 nS (variance 3829±851 pS) in the former and 13.4±4.0 nS (variance 4628±711 pS) in the latter. In neurones with TDIR, E_Bg_ was 3.1±0.9 nS (variance 1707±402 pS) and in non-TDIR neurones it was 2.9±0.9 nS (variance 2223±512 pS). None of these values differed between the groups or from the values in the pooled neurones. I∶E ratios were also very similar (5.0±0.7 v 4.4±0.9). Thus, it seems unlikely the differences in L2 compared to L3 and L5 are explicable in terms of heterogeneity of morphology/intrinsic properties in the L2 population.

Another potential confounding factor in comparing background synaptic conductances across layers could be the intrinsically generated theta oscillations reported to occur in L2 stellate neurones at depolarized potentials [48], [49], [50]. However, similar intrinsic oscillations have also been reported in L5 neurones, although not in L3 [51], [52], [53]. Whilst intrinsic theta oscillations do appear to be a characteristic of L2 neurones, in over 25 years of recording EC neurones, we have not seen such oscillations consistently expressed to the same extent as reported by others [48], [49], [50]. It does not appear to be present in all neurones that would be tentatively identified as stellate on the basis of TDIR, and, when detectable, can be weak and intermittent. Even if present, it should not influence our VmD estimations since Vm fluctuations are estimated on a considerably higher moment-to-moment time scale (in 10 ms bins) than that pertaining to the low frequency (7–8 Hz) intrinsic theta oscillations. Nevertheless, we looked at this as a possible confounding factor in our VmD estimations in L2. We subdivided the population of neurones with TDIR into those in which we could see evidence of theta oscillatory activity (n = 8) and those where we could not (n = 7). In the former, I_Bg_ was 12.2±4.6 nS, and in the latter, non-significantly different at 13.3±5.0 nS. Likewise, E_Bg_ was also very similar in the two populations (2.9±0.7 nS v 3.5±1.2 nS).

The oscillatory activity in L2 is principally dependent on a low-threshold, persistent Na- current and can be eliminated by Na-channel blockade [48], [49]. We recorded from L2 neurones with electrodes containing QX-314 (50 mM), to block the Na-currents (n = 5). We also substituted Cs-methanesulphonate (2 mM) for K-acetate in the recording solution, which eliminates TDIR [19], [47] (although the underlying I_h_-current does not play a prominent role in oscillatory activity [48]). We estimated conductances 4–5 minutes after impalement with these electrodes and the values obtained were well within the range of those seen with K-acetate electrodes (10.1±2.0 nS and 3.2±0.5 nS for I_Bg_ and E_Bg_, respectively). Fifteen minutes after impalement, Na-dependent action potentials had disappeared, and there was no evidence of TDIR, or of theta oscillatory activity at depolarized potentials. Respective values for inhibition and excitation at this time were 12.2±2.7 nS and 3.6±0.3 nS. Neither conductance was significantly altered when comparing the early and late time points and ratios remained remarkably similar (3.2 v 3.5). Thus, we are confident, that intrinsically generated oscillatory activity is unlikely to influence the comparison of VmD estimations in L2.

Background activity during acute network synchronizationBlocking GABA_A_r in cortical slices leads to progressive network synchronization culminating in emergent paroxysmal events that have often been used as a model of epileptiform activity. The EC is no exception and we have previously shown that such events are likely to originate in L5 and propagate from here to other layers of the EC and to other areas [18], [20], [21]. We have also shown that events in L2 are less frequent and not as pronounced as those in the L5 and we have suggested that the intrinsic properties of the superficial network may make them less susceptible to synchronization [20], [29]. We have used the VmD approach to chart the time course of changes in background inhibition and excitation that occur across L2, L3 and L5 in the period leading to overt synchronization of the synaptic networks. The intracellular recordings in the different layers were not conducted simultaneously but represent distinct data sets in different slices. As far as possible, matched experiments in slices from the same animal were conducted on the same day.

**Figure 5:**
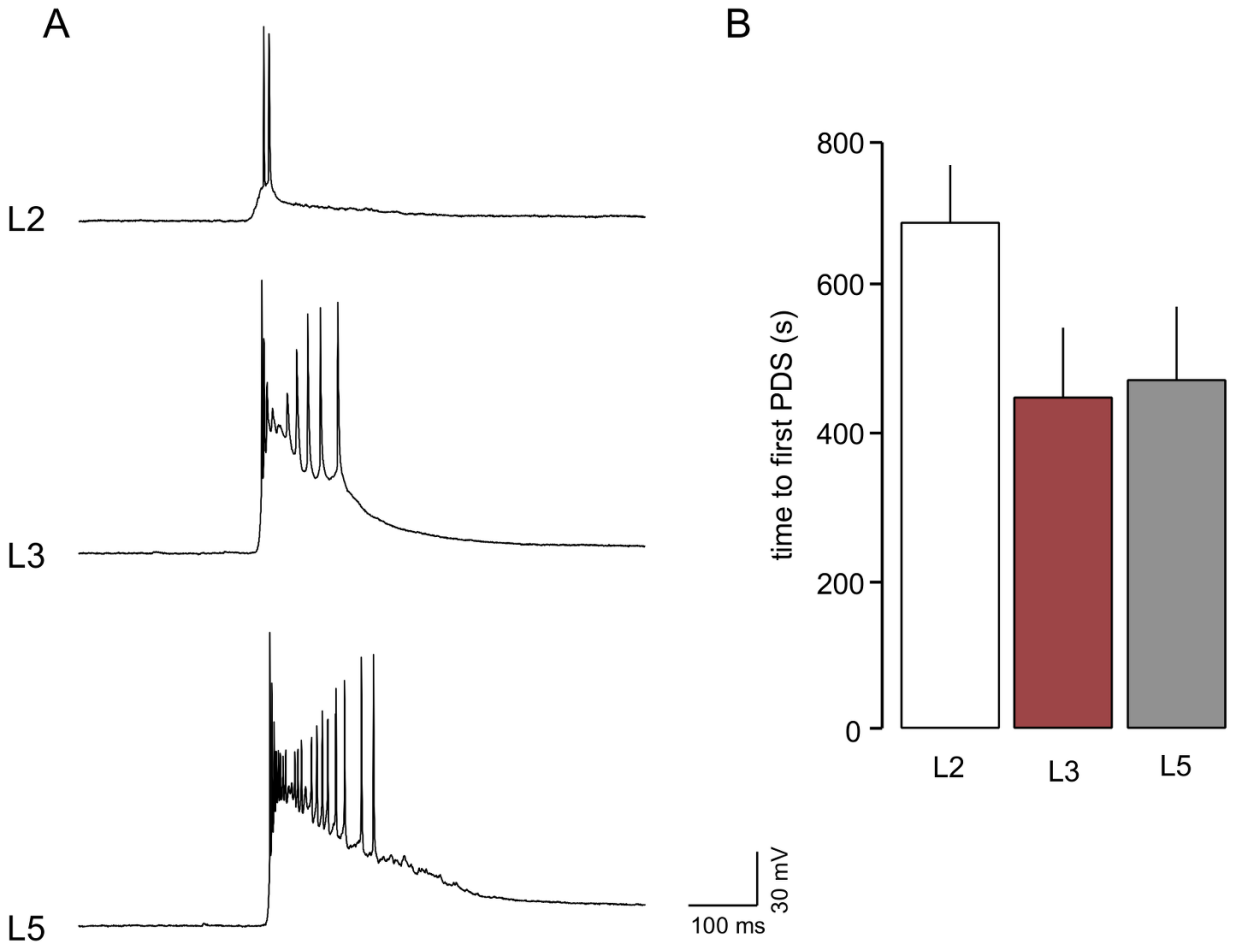
Bicuculline induced paroxysmal depolarizing shifts in EC neurones. A. Intracellular recordings show large depolarizing events associated with multiple spikes were recorded in both L5 and L3 during bicuculline (10 µM) perfusion. In L2 these were much smaller and often associated with just one or two spikes. B. In addition their appearance (timed from the entry of bicuculline into the bath) was delayed in L2 compared to the deeper layers.

Examples of intracellularly recorded, network-driven, paroxysmal depolarizing shifts in the different layers during perfusion with the GABA_A_r antagonist, bicuculline (10 µM), are shown in Figure 5A. For illustrative purposes these were actually recorded in the same slice (although not concurrently) after synchronous network activity had developed, and the events are typical of those arising during early network synchronization. They can vary somewhat between slices and preparations, but are fairly stereotypical in morphology. They are emergent network driven events as they correspond to population events recorded extracellularly at adjacent sites (not shown). Those in L3 and L5 were similar, and different in several ways from those in L2. Due to the complex nature of events it is difficult to quantify them meaningfully, but nominal values give some indication of differences. Peak amplitudes from resting potential (ignoring spikes) were almost the same in L5 and L3 (42±8 mV and 37±9 mV, respectively) but considerably and significantly greater (P>0.05) than those in L2 (16±6 mV). Duration was greatest in L5 (687±88 ms) followed by L3 (489±101 ms) and L2 (242±44 ms), and the number of spikes (full or truncated) associated with events showed a similar pattern (26±11 v 14±6 v 6±2). We measured the mean latency to appearance of the first spontaneous event after the entry of the antagonist into the bath (Figure 5B) and this showed that synchronized discharges appeared at around 450 s from initial exposure to bicuculline in both L3 and L5 but was significantly (P>0.05) delayed in L2 appearing around 700 s.

Thus, this comparison supports our previous studies [20] showing a greater susceptibility to disinhibition-induced synchronicity amongst L5 neurones compared to L2, and puts L3 intermediate between the two. We have now used the VmD approach to document how this may be related to changes in I∶E balance and excitability during the process of disinhibition. We have previously described some of this information for L3 neurones [40], and now present a fuller description with comparison to L5 and L2.

**Figure 6:**
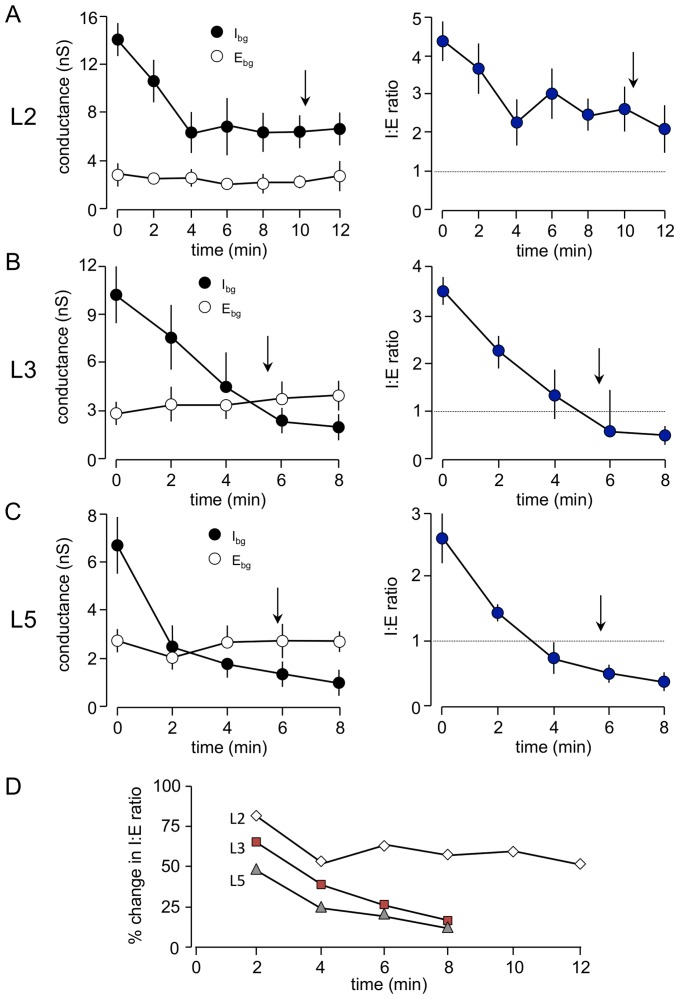
Time course of changes in global background synaptic conductances during perfusion with bicuculline. Bicuculline (10 µM) was appied at time 0. Arrows show the approximate time at which paroxysmal activity appeared. In all cases, once synchronized activity was established it became difficult to conduct further meaningful VmD measurements as the events increased in frequency and complexity. A. In L2 inhibition declined rapidly for the first 5–6 minutes before stabilizing at around 30–40% of control. Background excitation was largely unaffected. Despite this, the fall in estimated inhibition, the latter predominated throughout and the I∶E ratio never fell below 1. B. In L3 inhibition also fell rapidly over 5 minutes and continued to decline reaching a loss of around 85–90%. Simultaneously, excitation showed a gradual increase, although this did not reach significance. The combine effect was a sustained decrease in I∶E ration that reversed in favour of excitation around the time when paroxysmal activity appeared. C. Changes in background activity in L5 were similar to those in L3 except that the fall in inhibition was even more precipitous, and reached close to 100% in some cases. D. The time course of changes in I∶E ratio show that this occurred more rapidly in L5 than L3 but reached a similar end-point whereas that in L2 never reached the same level as that in either of the deeper layers.

Figure 6C illustrates the time course of changes in background conductances during perfusion with bicuculline (10 µM) in L5 neurones (n = 6). As expected, there was a rapid decrease in I_bg_ during the first 2 minutes of contact with the antagonist, concurrent with a small, but non-significant, decrease in E_bg_. At this stage the absolute levels of the conductances were approximately equal. Thereafter, I_bg_ continued to decline whereas E_bg_ returned to its original level, with the absolute magnitude of the conductance levels reversing between 2 and 4 minutes. The net effect of these changes was a substantial switch in I∶E ratio to favour excitation compared to the bias towards inhibition under control conditions. Results for L3 neurones (Figure 6B) were similar, but the decline in I_bg_ was less rapid and the reversal of conductance levels occurred a little later (around 5 min). I∶E ratio in L3 neurones also reversed in favour of excitation, and, although the change was slightly less rapid, it reached the same end point as in L5 and the time of appearance of paroxysmal activity occurred when the I∶E ratio reached approximate equivalence (Figure 6D).

**Figure 7:**
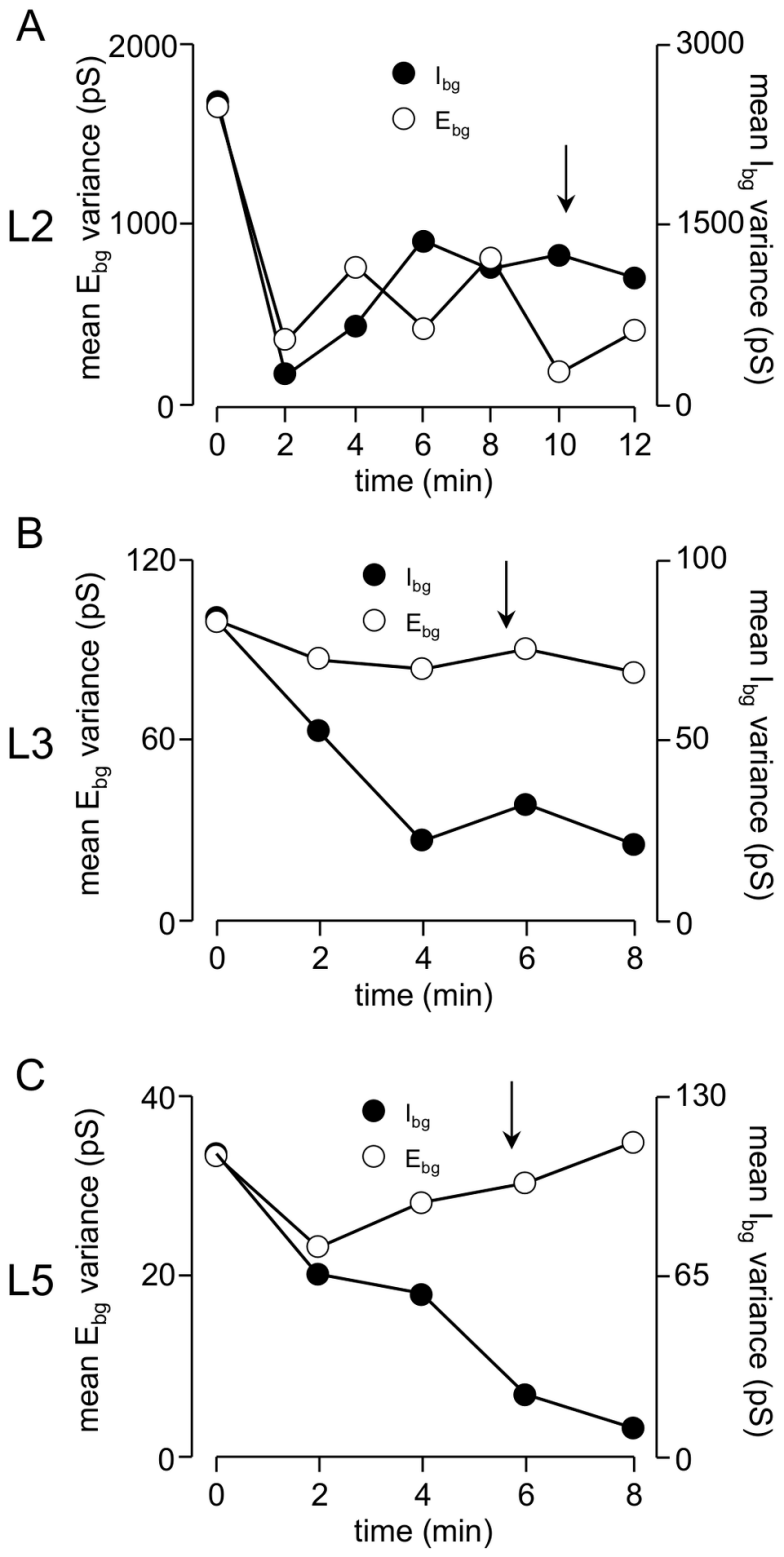
Changes in variance of background conductances during bicuculline perfusion. There were substantial differences in variances between and within layers, but scales have been adjusted to allow for comparison of the time course of changes. A. In L2 the variance (and thus synchronicity) of both inhibition and excitation fall rapidly over 2–3 minutes and thereafter remained reasonably stable at the lower level, although that of inhibition tended to increase again after the first fall. B. In L3 variance of inhibition fell steadily and remained stable whereas that of excitation showed little change. C. Similar to L3, inhibition variance fell throughout in L5, but an initial fall in variance of excitation was followed by a clear recovery to control levels. Again, the arrows indicate the approximate time at which paroxysmal activity appeared.

Results for L2 neurones (n = 6) are shown in Figure 6A. Again, there was an initial rapid fall in I_bg_. However, after 4 min, this had plateaued at around 45% of control, and thereafter there was no further substantial fall. E_bg_ showed little concurrent change, and the absolute values of the conductances did not reverse at any point. Consequently, although the I∶E ratio fell by around 50%, it always remained substantially in favour of inhibition (Figure 6C,D). Thus, the delayed appearance of spontaneous synchronized events and, perhaps, the extent of synchronization in L2 could be related to the amount of residual inhibition. We have also monitored the variance of the background conductances throughout application of bicuculline (Figure 7). During bicuculline perfusion, the variance of E_bg_ initially fell quite steeply and then essentially plateaued. Perhaps not surprisingly, the variance of I_bg_ fell precipitously, but then actually increased towards control levels. In L3, the variance of I_Bg_ fell markedly but that of E_Bg_ was largely unaltered. Likewise, in L5 the variance of I_bg_ fell progressively throughout, whereas, after an initial fall, there was a delayed increase in E_bg_ variance taking it beyond control levels.

Finally, we noted small changes in intrinsic neuronal excitability in all three layers during perfusion with bicuculline. In L2, spike threshold fell slightly from 18.8±0.4 mV (positive to rest) to 17±0.7 mV, and in L3 from 20.6±18.2 mV to 18.2±0.9 mV, although neither change reached significance. In L5, threshold was reduced from 24.2±0.5 mV to 20.7±0.8 mV, and this was significant (*P*<0.05). However, it should be noted that the normalized change was very similar in all three layers (around 9 to 10%). Concurrently, the number of spikes evoked by a long depolarizing pulse was slightly increased in all layers (L2: 4.0±0.8 v 5.7±0.6; L3: 4.3±0.3 v 5.5±1.0; L5: 2.7±0.2 v 3.3±0.9) but none of the changes reached significance. The normalized changes were again similar in all layers (around +13%). Thus, there were indications that the loss of background synaptic inhibition increased intrinsic excitability *per se* (cf [39]), and this could contribute to an overall increase in network excitability.

An important question concerns the origin of the residual inhibition in L2. Our patch clamp studies showed that glycine receptors were likely to make a minor contribution to spontaneous inhibition in L2, although the VmD analysis did not show a significant fall in I_bg_ with strychnine. Nevertheless, we thought it worthwhile to examine whether strychnine could enhance the epileptogenic effects of bicuculline in L2. In 4 slices where bicuculline failed to elicit synchronous discharges in L2, subsequent addition of strychnine (up to 4 µM) failed to provoke the appearance of such activity. In a further 4 slices where brief discharges were present, these were unaffected by strychnine (1–2 µM). However, in 2 slices, brief synchronized discharges appeared to be enhanced by strychnine (1 µM; not shown), a weak effect that reversed on washing out the antagonist. To attempt to further block the residual inhibition in L2 we combined bicuculline and strychnine with the non-competitive blocker, picrotoxin (n = 4). Fifteen minutes after perfusion with bicuculline and strychnine E_bg_ was essentially unaltered compared to control (4.1±1.1 nS v 4.5±1.2 nS). I_Bg_ was concurrently reduced from 13.9±3.9 nS to 6.3±2.7 nS, similar to or slightly greater than to the fall seen with bicuculline alone (see above). Subsequent addition of picrotoxin saw a further fall in I_Bg_ to 4.9±2.3 nS (*P*<0.05). Overall, the changes reflect a fall in I∶E ratio from 4.4±1.1 in control to 1.5±0.6 with all 3 antagonists present, and show that the combined blockers resulted in a greater decline in inhibition (by approximately 60%) than with bicuculline alone (40–50%), although the decrease was still less than that seen with bicuculline alone in either L3 or L5.

**Figure 8:**
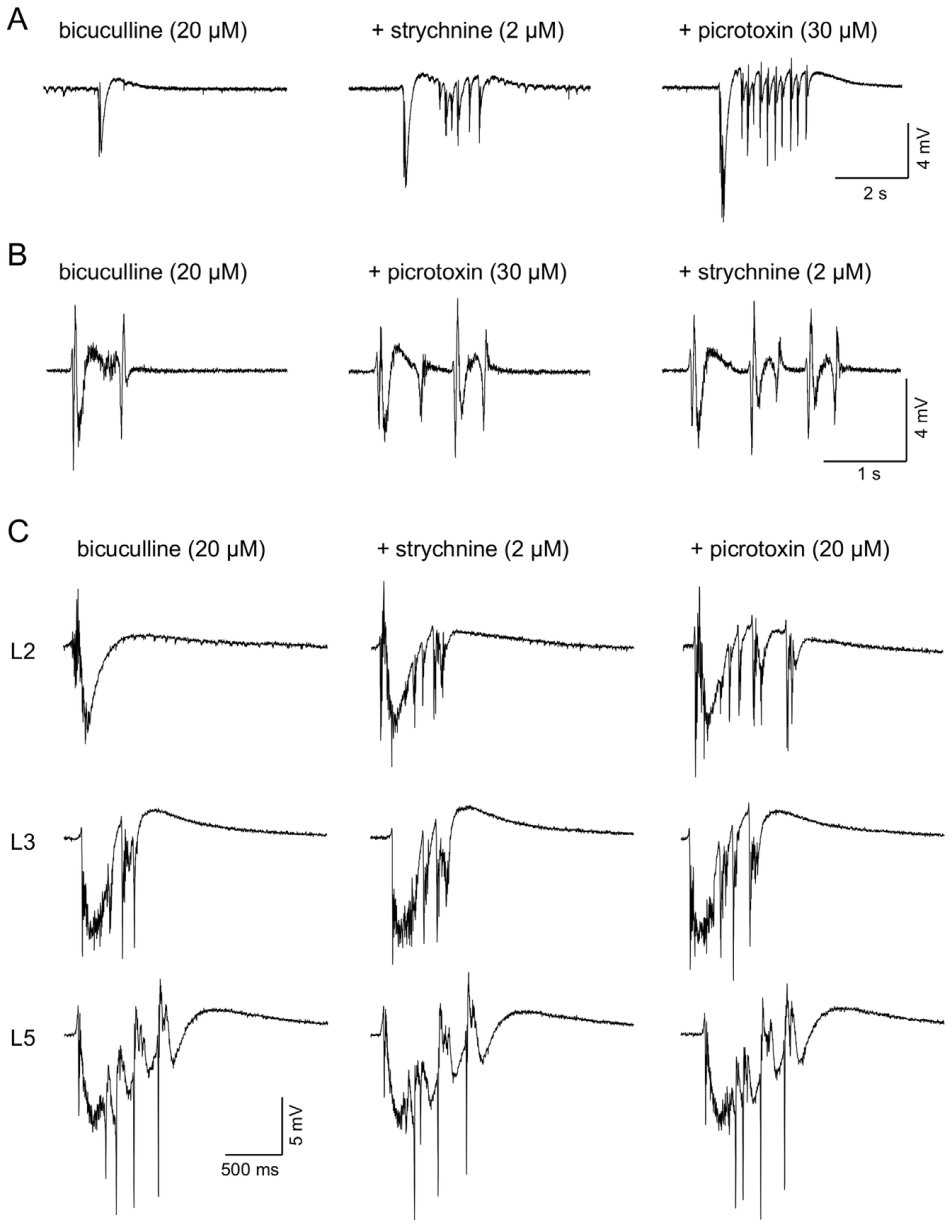
Additional effects of strychnine and glycine on paroxysmal activity in L2. A. In the presence of bicuculline the spontaneous activity recorded in L2 consisted of single brief negative-gong events. Following addition of strychnine this was joined by a series of brief afterdischarges. A further, and more pronounced, exacerbation of the activity was seen when the non-competitive GABA_A_r antagonist, picrotoxin was added to the cocktail. B. A similar study in L2 of another slice shows that the same result was obtained when the order of application of strychnine and picrotoxin was reversed. C. In this slice we made simultaneous recordings at locations in L2, L3 and L5. Spontaneous events in bicuculline were briefer and less complex than those seen in the deeper layers (cf. Fig. 5). Addition of strychnine and then picrotoxin, as in the other studies increased the duration and complexity of events in L2, but left those in L3 and L5 largely unaltered.

The combination of blockers did appear to be associated with an increased severity of epileptiform discharges in L2 (n = 6). This is illustrated Figure 8. In these slices bicuculline-induced discharges were already present, and we applied the other blockers sequentially and cumulatively, and compared the epileptiform activity induced, using extracellular recording. We found that addition of strychnine resulted in a weak enhancement of the discharges in three slices and these were then further enhanced by picrotoxin. In one slice strychnine had little detectable effect, but the discharges were enhanced by picrotoxin. In two other slices picrotoxin was applied first and enhanced the discharge; subsequently, strychnine further enhanced the discharge in one but had little effect in the other. When simultaneous recordings were made in other layers, we saw little change in the local discharges when picrotoxin and strychnine were added to bicuculline.

## Discussion

Patch clamp recordings of EPSCs in L3 revealed a greater degree of spontaneous excitation compared to L2 and L5. This was mainly reflected by a substantially higher frequency of sEPSCs. Interestingly, the frequency of mEPSCs was very similar in all 3 layers, indicating that activity dependent release driven by action potentials was considerably higher in L3. Using paired intracellular recordings we have previously shown a relatively high incidence of single axon connections between principal cells in L3 [46]. It is possible that the greater frequency of sEPSCs seen in our patch clamp recordings reflects a high level of spontaneous firing in recurrent axon collaterals between L3 pyramids. What may also be significant is that spontaneous firing rates of L3 neurones *in vivo* is generally higher than those in L5 or L2, which could contribute to greater recurrent excitation in the middle layer [6]. In addition, we have provided evidence for a high degree of electrical coupling between L3 neurones [46], which could reinforce a high level of recurrent excitation. There was a similar incidence of recurrent excitatory connections in L5 and L3, although the recurrent EPSPs were considerably smaller in the deeper layer [46], but a lower neuronal firing rate could be a factor in a lower recurrent excitation and hence lower frequency of sEPSCs. Interestingly, Quilichini *et al.*, [6] found no correlation between the firing rate of neurones and intrinsic excitability suggesting that the former was dependent on the network connectivity rather than biophysical characteristics.

In previous patch clamp recordings we demonstrated that spontaneous GABA release in L2 was dramatically higher than in L5 [37]. The current experiments show strong similarity between L2 and L3 in this respect, with a high level of baseline release. However, in L3, action potential driven IPSCs (around 50%) accounted for a much higher proportion of baseline release than in L2, where sIPSC and mIPSC frequencies were almost the same. Thus, a patch-clamp comparison of spontaneous glutamate release in the three layers shows that levels are highest in L3, with L5 next, and L2 lower than L5, but not dramatically so. In contrast, spontaneous GABA release is highest in L2, with L3 similar but L5 dramatically lower than either. Monitoring the frequency of spontaneous currents can be misleading and probably tells us little about the level of functional background inhibition or excitation. Estimating arbitrary charge transfer levels associated with spontaneous currents over time may give us a better picture of this, and these estimations suggested a dominance of inhibition over excitation in all three layers, with a rank order of I∶E ratio L2>L3>L5. However, this comparison gives an approximate view of the situation at best because the somatic whole cell patch clamp recordings assess inhibitory and excitatory currents in isolation, and are also likely to exclude many events occurring at distal dendritic sites. Nevertheless, it does give a *relative* picture of what the neurones see from their presynaptic inputs in terms of excitatory and inhibitory network activity.

To obviate some of the limitations associated with the patch clamp recordings, and to align the presynaptic activity with what the neurone undergoes postsynaptically, we made VmD measurements [42] to estimate global background excitation and inhibition. This has a number of advantages: it specifically estimates conductances mediated via AMPAr activation and GABA_A_r activation, which are, overwhelmingly, the source of background postsynaptic excitation and inhibition, respectively; inhibitory and excitatory conductances are estimated concurrently in the same neurone, and essentially reflect network input to all compartments (somatic, dendritic) integrated across the whole neurone; within limitations, the variance of the conductances may partly reflect the degree of correlation between presynaptic inputs, and hence, the state of the network; sharp electrode recordings permit simultaneous measures of neuronal excitability.

VmD estimations of postsynaptic conductances generally aligned with the presynaptic activity monitored by the whole cell patch clamp experiments, although relative differences were less pronounced. Thus, E_bg_ was approximately equal in L2 and L5, and, although higher in L3, not dramatically so. I_bg_ was highest in L2, lower in L3, but considerably lower in L5 compared to the more superficial neurones. In agreement with CT estimations from the patch clamp recordings, I∶E ratios from VmD estimations again showed a dominance of inhibition over excitation in all three layers, and the rank order was the same, albeit with a somewhat reduced difference between L3 and L5. This suggests that the dominant effect of on-going presynaptic network activity in all layers in our slices is inhibition. Although the brain slice, with its reduced synaptic network, reflects a generally quiescent situation, there is qualitative agreement with the situation *in vivo*. For example, VmD estimations from *in vivo* intracellular recordings in parietal cortex (layer not specified) of un-anaesthetized cats [54], show that an inhibition-dominant network situation is extant during both the awake state, and during natural sleep, although the I∶E ratio in the latter was higher in the former (approx. 3.9) compared to the latter (approx. 2.6). Interestingly, the I∶E ratio during wakefulness is similar to that seen in L3 and L5 neurones in our experiments, whilst that in L2 approximated the sleep state in vivo. It would be a huge leap of imagination to propose that the networks in L5/L3 are in an “awake state” whereas that in L2 is “sleeping”, but the data does suggest that network excitability in L2 and, thus, afferent transfer to the hippocampus, is very tightly controlled by synaptic inhibition.

It has been suggested that the variance of postsynaptic background synaptic conductances may give an estimate of the average correlation of inhibitory or excitatory activity in the presynaptic inputs, with a high variance predicting some degree of synchronization [42]–[44]. It should be noted that this prediction relates to the in vivo situation where transmitter release rates are likely to be much higher than in our slices. Examination of the variances across layers in our slices reveals that the most obvious difference was the high degree of variance in both E_bg_ and I_bg_ in L2 compared to L3 and L5. If we make the assumption that conductance variance provides a measure of synaptic correlation, even under the relatively quiescent conditions in the slice, this could be taken as evidence that there is some degree of synchronization in the network of L2. This may be supported by observations that the power of theta, gamma and high-frequency oscillations, which could reflect synchronized network activity, is high in superficial layers compared to the deeper layers [5], [6], [55], [56].

If we accept that that there may be some correlated activity in L2 synaptic inputs, it is possible to speculate that such a property could be related to the level and organization of synaptic inhibition. It is clear that number, diversity, and synaptic connectivity of inhibitory neurones is progressively greater as we move from L5 through L3 to L2 of the EC [57]–[69], and this is reflected by functional measures of inhibition (present study; [37], [38]). Network synchrony can be driven by interneurone-interneurone interactions and/or interneurone/principal cell interconnectivity [70]–[73]. Thus, it is possible that the high variance of I_bg_ in L2 is at least partly due to correlated release from GABA inputs resulting from interneuronal interactions with principal neurones and other interneurones. Using paired cell recordings we demonstrated a paucity of recurrent connectivity between principal neurones in L2 [46]. This was confirmed by a recent study [74], which also showed that the high level of inhibition in L2 is congruent with extensive interconnectivity of principal neurones via interneurones. We previously demonstrated that basket-like interneurones in L2 have widespread axonal arbors restricted to the layer and have a powerful NMDAr mediated excitatory drive [75]. A reduction in the number of these neurones or blockade of the NMDAr-mediated drive onto them disrupts gamma oscillations, which may depend on synchrony within the principal neurone-interneurone connectivity [76].

It has long been accepted that synaptic inhibition is able to “balance” and control neuronal excitation. Trevelyan *et al.*, [77] provided a very clear demonstration of how emergence of network synchronization during epileptiform activity is restrained by on-going synaptic inhibition. In our studies, reducing background inhibition by blocking GABA_A_R resulted in a rapid synchronization in L5 and the emergence of interictal-like epileptiform activity. Despite a much higher level of background inhibition, the result was almost identical in L3, and this could be associated with the higher initial E_bg_ in this layer, suggesting that the inhibition-excitation balance is crucial in restraining network synchronization. Paradoxically, L2, which may already have a degree of correlative activity in its synaptic networks, was relatively resistant to emergent network synchronization when inhibition was pharmacologically reduced with bicuculline. Emergent synchronization was delayed, was less marked than in other layers, and was not seen in some slices that did display such activity in other layers.

Examination of the changes in I_bg_ and E_bg_ during bicuculline perfusion revealed that the former fell rapidly and network synchronization emerged around the time when its absolute value was exceeded by the latter, in both L5 and L3. However, this did not occur in L2 and normalized data showed that I_bg_ was reduced by only 50–60% compared to 80–90% in the deeper layers. Residual inhibition in L2 could have a source other than GABA_A_r activation, and one possibility is that it could be mediated by glycine. There is evidence for expression of glycine receptors in the EC with the β-subunit, in particular, apparently localized to the superficial layers [78] and glycine elicits strychnine-sensitive inward currents in L2 neurones [79]. sIPSC frequency is weakly reduced by strychnine in L2 but not L5 (present study; [37]). The VmD approach will not readily distinguish between GABA and glycine as the reversal potentials for the two are essentially the same, but we did not find any overwhelming evidence that glycine receptors make a major contribution to the residual inhibition in L2. I_bg_ showed a very small, but non-significant fall during blockade of receptors with strychnine. Although we occasionally found that strychnine appeared to enhance the synchronizing effects of GABA_A_r blockade in L2, in the majority of cases it had no such effect. Interestingly, glycine receptors in L2 can be desensitized by GABA but the reverse is not true [79]. Thus, it is possible that glycine inhibition may actually become more effective as GABA-inhibition is reduced by bicuculline, but this was not really supported by the failure of strychnine to consistently enhance the effects of bicuculline. Finally, strychnine also slightly reduced sIPSCs in L3 as well as L2, but residual inhibition was not evident in in VmD estimations in the former.

An alternative explanation for residual inhibition is that very high levels of ambient GABA associated with spontaneous release are able to compete with the bicucullne to maintain the level of spontaneous inhibition. Addition of the non-competitive GABA_A_r blocker, PTX did reduce I_Bg_ to a greater extent than bicuculline alone, although there was still around 30–35% residual inhibition in L2. Thus, the source of the residual inhibition in L2 is still a matter for debate, but what seems clear is that the overall “inhibitory veto” [70] is much stronger in this layer than in L3 and L5.

The significance of our results for the overall functional roles of the EC in physiological processes is a matter for speculation at present. Nevertheless, it is clear that the network properties across layers support very different processing roles and capabilities. For example, as mentioned above, L2 neurones express prominent grid cell properties that contribute to a vital role in spatial navigation. Grid firing is suggested to be generated within the recurrent inhibitory microcircuitry of this layer rather than dependent on recurrent excitatory interactions [74]. Our current, and previous, observations [38], [46], [75] support the suggestion of predominance of recurrent inhibition in this layer and an inherent rhythmicity in this network that could help define grid cell activity. The powerful background synaptic inhibition may be crucial in this regard. Destexhe [44] has argued that spike firing and patterning in cortical networks is determined by fluctuations in background inhibitory “noise”. The high level of background inhibition, coupled with a low level of background excitation could support a crucial role in determining grid cell patterning. Varying ratios could be deterministic in different firing patterns and functionalities in other layers.
